# Atrial Septal Aneurysm and Atrial Septal Defect Association - an Uncommon But Well-Recognized Association

**DOI:** 10.21470/1678-9741-2020-0464

**Published:** 2021

**Authors:** Antonio Carlos Menardi, Paulo José de Freitas Ribeiro, Paulo Roberto Barbosa Evora

**Affiliations:** 1 Division of Cardiovascular and Thoracic Surgery, Department of Surgery and Anatomy, Ribeirão Preto School of Medicine, University of São Paulo, Ribeirão Preto, São Paulo, Brazil.; 2 CECORP - Specialized Heart and Lung Center, Ribeirão Preto, São Paulo, Brazil.

**Keywords:** Heart Septal Defects, Atrial, Heart Aneurysm, Cardiopulmonary Bypass, Hemodynamics

## Abstract

Atrial septal aneurysm (ASA) is an uncommon but well-recognized cardiac abnormality. This educational text reviews the case of a 54-year-old female presenting an ASA related to a small ostium secundum atrial septal defect. The considerable signs and symptoms, interestingly, have not been justified by the clinical and hemodynamic investigations. So, we opted for a better imaging investigation with cardiac catheterization and transesophageal echocardiography. The surgical process was earlier indicated and performed with aid of cardiopulmonary bypass.

**Table:** 

Abbreviations, acronyms & symbols	
**AN** **ASA** **ASD** **CPB** **LA** **LV** **PFO** **RA** **RV**	**= Aneurysm** **= Atrial septal aneurysm** **= Atrial septal defect** **= Cardiopulmonary bypass** **= Left atrium** **= Left ventricle** **= Patent foramen ovale** **= Right atrium** **= Right ventricle**

## INTRODUCTION

**Fig. 1:**
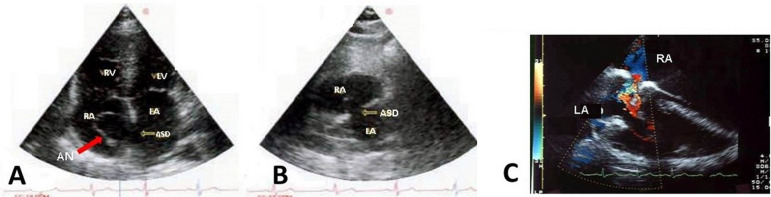
Transthoracic echocardiogram. A) The red arrow points to the atrial septal aneurysm; B) the arrow points to the atrial septal defect (ASD); and C) the echo Doppler shows the ASD flow. AN=aneurysm; LA=left atrium; LV=left ventricle; RA=right atrium; RV=right ventricle.

**Fig. 2:**
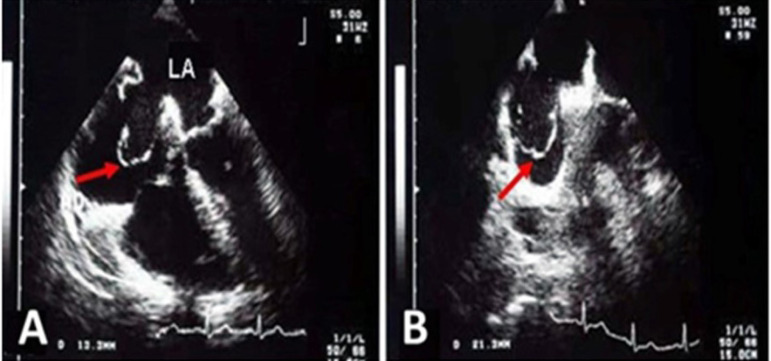
Transesophageal echocardiogram. A and B) The red arrow points to the atrial septal aneurysm. LA=left atrium.

Atrial septal aneurysm (ASA) is an uncommon but well-recognized cardiac abnormality. This educational text reviews the case of a 54-year-old female presenting an ASA related to a small ostium secundum atrial septal defect (ASD) (Figure 1). The considerable signs and symptoms, interestingly, have not been justified by the clinical and hemodynamic investigations. So, we opted for a better imaging investigation with cardiac catheterization and transesophageal echocardiography. Heart catheterism revealed a 13-mm orifice that was once evidenced by transthoracic echocardiography, but with an insignificant shunt and without pulmonary hypertension. The transesophageal echocardiography (Figure 2) showed a membrane entering and leaving the atrial chambers. Chest X-ray showed standard cardiac dimensions and clear lungs. And the electrocardiogram showed atrial fibrillation with a ventricular rate of 90/min, ordinary QRS, and T waves. In the transthoracic echocardiography, right ventricular overload was evident, as suggested by the ASA description (Figure 1).

QUESTIONS - Is ASA and ASD a frequent association? What are the definitions of ASA?
- Which are the main manifestations attributed to ASA?
- Regarding the contemporary imaging techniques, which would be the most adequate for the prognosis of ASA?
- What would be viewed as clinical challenges?
- What would be viewed as stroke challenges?
- What about the ASA association with migraine?
- What would be viewed as surgical challenges?

### Discussion of Questions

**Fig. 3:**
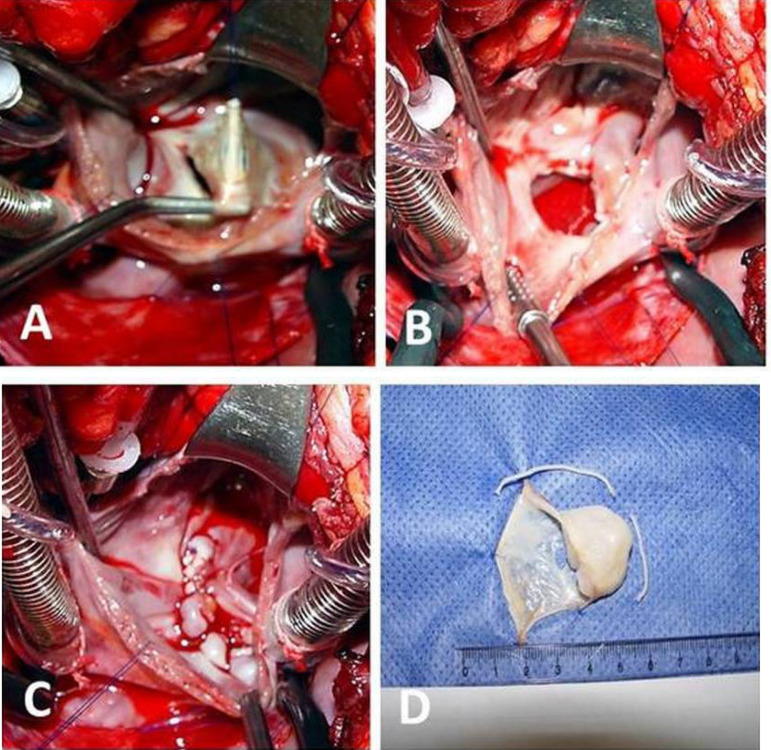
Surgical images. A) Atrial septal aneurysm (ASA) pulled up through the atrial septal defect exposed by a right atriotomy; B) fossa ovalis after ASA excision; C) atrium septorhaphy; D) excised aneurysm.

(Question A) The fact that ASA association with ASD is an uncommon cardiopathy is evidenced by Ruiz de Larrea C. et al. ^[^^1^^]^. (1993) These authors reported only twelve (0.22%) cases among 5,221 two-dimensional echocardiographies in cardiopaths ^[^^1^^]^. However, it should be a contributing aspect to cardioembolic stroke.

(Question B) Although precise ASA definitions differ according to measurement ^[^^2^^-^^4^^]^ and stage (mobility) ^[^^5^^,^^6^^]^, ASA is a "saccular" deformity, commonly located at the fossa ovalis, protruding to the right or the left atrium or on each aspect ^[^^2^^]^. An ASA may be isolated or associated to any other anomaly. The most well-known affiliation is the patent foramen ovale (PFO).

(Question C) The essential manifestations are atrial arrhythmias and arterial embolism. An interatrial septal aneurysm can act as an arrhythmic focus, generating focal atrial tachycardias. Also, ASA's presence tends to irritate the stasis of left atrial blood flow and predispose to left atrial clots and systemic thromboembolism ^[^^7^^]^. Right to left shunting, or the aneurysm itself, must be the mechanism of cardioembolic stroke.

(Question D) A transesophageal echocardiography is more sensitive in ASA diagnosis than a transthoracic echocardiography ^[^^4^^,^^6^^,^^8^^]^. Magnetic resonance and cardiac computed tomography imaging are additionally useful for the diagnosis of ASA ^[^^9^^,^^10^^]^.

(Question E) The most relevant is the arterial embolism that can cause a catastrophic stroke. Atrial arrhythmias can predispose to arterial embolism. It is mandatory to emphasize the ASA function in cerebral embolism. Although atrial fibrillation is a common cause of stroke, the ASA presence could be contributory, and it is of some interest because it may worsen strokes. Therefore, anticoagulation and viable surgery selections are fundamental challenges. The anticoagulation choice is a matter of controversy. Aspirin was used solely to prevent thromboembolism due to anticoagulation's logistical difficulties with warfarin. The patient chose antiplatelet tablets and preferably oral anticoagulation for secondary prevention of cardioembolic events. The efficacy of aspirin is advised by the French PFO-ASA study; in 216 people suffering with cryptogenic stroke and PFO alone, recurrent stroke on aspirin was once 2.3% after four years, comparing to 4.2% in sufferers with neither PFO nor general ASA ^[^^11^^]^.

(Question F) According to Mas et al. ^[^^11^^]^ (2001), ischemic stroke in young patients is associated in up to 30% of cases with PFO with or without ASA. A frequent association between migraine and PFO has been described, but few studies have addressed the possible association between ASA and migraine in stroke patients. In patients with ischemic stroke and PFO, the probability of having ASA could be higher in migraine sufferers ^[^^12^^]^. This finding could have diagnostic implications, suggesting the convenience of seeking this association in these patients.

(Question G) Besides the chance of embolism, the patient was once operated on due to functional symptoms, no longer justifying imaging and hemodynamic investigations. The surgical process was earlier indicated and performed on January 11, 2006, with cardiopulmonary bypass (CPB). Membrane exeresis (Figures 3A and 3B) and running suture of the ostium secundum were carried out. Also, a tricuspid valve cerclage annuloplasty was performed. Atrial dilatations and the presence of an atrial defect and a multiperforated membrane with saccular redundancy shape were the critical intraoperative observations. The excised aneurism is showed in Figure 3D. The surgical procedure was uneventful, with an anoxia time of 45 min, CPB time of 55 min, and surgical procedure time of 150 min. The extracorporeal circulation discontinuation was uneventful, which includes the evolution in the intensive care unit and infirmary. The patient was discharged on the 4^th^ postoperative day in an exceptional situation, and her ten-year follow-up shows a notable outcome.

## BRIEF CONSIDERATIONS OF THE CASE REPORTED

ASA is rare and is frequently an unintentional finding. Previously recognized on postmortem only, it is now evidenced on echocardiography or by comparing ischemic stroke images. Congenital malformation of the atrial septum contributes to increase the ASD, as it was suggested by Hanley PC et al. ^[^^2^^]^.

1. Uncomplicated and isolated ASA requires no specific treatment other than the follow-up. Patients should be evaluated for the presence of thrombus in the aneurysm. Options for preventing recurrent stroke include medical therapy with antiplatelet or anticoagulant agents or surgical or percutaneous closure.
2. However, in the presence of a shunt, it is preferable to close it to prevent recurrent paradoxical embolism. The transcatheter procedure is now safe and effective, and commonly used for this purpose, even though the superiority of closure over the best medical therapy has not been established.

## LEARNING POINTS

- Uncomplicated and isolated atrial septal aneurysm (ASA) requires no specific treatment other than follow-up. Patients should be evaluated for the presence of thrombus in the aneurysm.
- Therapeutic options for prevention of recurrent stroke in patients with ASA as well as an atrial septal abnormality - including patent foramen ovale (PFO), ostium secundum atrial septal defect (ASD) - are medical therapy with antiplatelet agents or anticoagulants and surgical or percutaneous closure of the defect.
- An anticoagulation choice is a matter of controversy. The patient chose antiplatelet tablets and preferably oral anticoagulation for secondary prevention of cardioembolic events.
- The French PFO-ASA study suggested aspirin therapy's efficacy in 216 patients with cryptogenic stroke and PFO alone. Recurrent stroke on aspirin was 2.3% after four years, comparing to 4.2% in patients with neither PFO nor ASA.

**Table:** 

Authors' roles & responsibilities	
ACM	Substantial contributions to the conception and design of the work; and analysis and interpretation of data for the work; revising the work critically for important intellectual content; final approval of the version to be published
PJFR	Revising the work critically for important intellectual content; final approval of the version to be published
PRBE	Substantial contributions to the conception and design of the work; and analysis and interpretation of data for the work; drafting the work or revising it critically for important intellectual content; final approval of the version to be published
